# Molecular markers associated with the outcome of tamoxifen treatment in estrogen receptor-positive breast cancer patients: scoping review and in silico analysis

**DOI:** 10.1007/s12672-021-00432-7

**Published:** 2021-10-01

**Authors:** Maiquidieli Dal Berto, Giovana Tavares dos Santos, Aniúsca Vieira dos Santos, Andrew Oliveira Silva, José Eduardo Vargas, Rafael José Vargas Alves, Fernanda Barbisan, Ivana Beatrice Mânica da Cruz, Claudia Giuliano Bica

**Affiliations:** 1 Laboratory of Pathology, Federal University of Health Sciences of Porto Alegre (UFCSPA), 245,Sarmento Leite street, Porto Alegre, RS 90050-170 Brazil; 2 Institute of Biological Sciences, University of Passo Fundo (UPF), 285, Brazil Avenue, Passo Fundo, RS 99052-900 Brazil; 3 Department of Clinical Medicine, Federal University of Health Sciences of Porto Alegre (UFCSPA), 245, Sarmento Leite street, Porto Alegre, RS 90050-170 Brazil; 4 Graduate Program in Gerontology, Federal University of Santa Maria, Santa Maria, RS 97105-900 Brazil; 5 Department of Basic Health Sciences, Federal University of Health Sciences of Porto Alegre (UFCSPA), 245, Sarmento Leite street., Porto Alegre, RS 90050-170 Brazil

**Keywords:** Recurrence, Tamoxifen, Breast cancer, HR positive, Biological processes, Molecular targets

## Abstract

Tamoxifen (TMX) is used as adjuvant therapy for estrogen receptor-positive (ER+) breast cancer cases due to its affinity and inhibitory effects. However, about 30% of cases show drug resistance, resulting in recurrence and metastasis, the leading causes of death. A literature review can help to elucidate the main cellular processes involved in TMX resistance. A scoping review was performed to find clinical studies investigating the association of expression of molecular markers profiles with long-term outcomes in ER+ patients treated with TMX. In silico analysis was performed to assess the interrelationship among the selected markers, evaluating the joint involvement with the biological processes. Forty-five studies were selected according to the inclusion and exclusion criteria. After clustering and gene ontology analysis, 23 molecular markers were significantly associated, forming three clusters of strong correlation with cell cycle regulation, signal transduction of proliferative stimuli, and hormone response involved in morphogenesis and differentiation of mammary gland. Also, it was found that overexpression of markers in selected clusters is a significant indicator of poor overall survival. The proposed review offered a better understanding of independent data from the literature, revealing an integrative network of markers involved in cellular processes that could modulate the response of TMX. Analysis of these mechanisms and their molecular components could improve the effectiveness of TMX.

**Supplementary Information:**

The online version contains supplementary material available at 10.1007/s12672-021-00432-7.

## Introduction

Breast cancer (BC) is the most prevalent cancer among women in developed and developing countries [1]. Due to its incidence and mortality, it is considered a serious public health problem worldwide [2, 3]. This type of cancer is a complex and heterogeneous disease with several distinct histopathological and molecular subtypes. Among them, about 60 to 70% of all diagnosed BC are positive for hormone receptor expression (HR+), specifically estrogen receptor (ER+) and/or progesterone receptor (PR+).

The stimulation of both receptors by its ligand molecules—estrogen and progesterone—induces mainly transcriptional changes, which trigger biological processes such as cell survival, proliferation, and differentiation. Therefore, in breast cancer, its constant stimulation by sexual hormones could contribute to tumor increasing and disease progression [4, 5], justifying anti-estrogen therapies as an adjuvant treatment for this type of neoplasia [6].

Tamoxifen (TMX) is one of the most used types of adjuvant treatment in ER+ breast cancer. Administered as a prodrug, TMX must be metabolized in the liver to form active metabolites that will perform their estrogen antagonist functions and thus referred to as a *selective estrogen receptor modulator* (SERM) which blocks the binding of estrogen to the estrogen receptor and consequently inhibit cell proliferation and survival [7–11]. For this reason, TMX is the standard hormone therapy for HR+ breast cancer patients in the premenopausal stage [12–14].

Whereas this hormone adjuvant therapy has considerably improved survival in HR molecularly subtyped BC patients in recent decades, the development of pharmacological resistance has been an increasing challenge for oncology, once the lack of responsiveness to treatment is a critical and limiting factor for therapeutic efficacy [15, 16]. Around 30 to 50% of HR+ BC patients, regardless of the expression level of these receptors, presented intrinsic or acquired resistance to TMX [17, 18]. Consequently, the five-year survival rate after resistance occurrence was less than 20% [19, 20].

Several clinical works have been published in the literature bringing information about a new specific marker involved with this frame of resistance, associating its molecular profile with a better or poor outcome in BC patients. Therefore, a better comprehension of the interrelationship among these identified molecular markers associated with TMX resistance becomes increasingly relevant and necessary to strengthen the understanding of what molecular mechanisms and cell processes are changed in tumor cells when they acquire hormone resistance in an attempt to overcome it and improving therapeutic effectiveness.

A major meta-analysis carried out by Wirapati et al. analyzed the gene expression in breast cancer patients and associated it with important biological processes that could be present in all molecular subtypes [21]. Another meta-analysis by Mihály et al. investigated new independent biomarkers that could be related to the tamoxifen response in breast cancer patients [22].

This work presents a systematic review of recent papers in the literature, focusing only on translational studies with a minimum 3-year follow-up. It highlights how individual biomarkers related to TMX resistance impact the survival rate of ER+ breast cancer patients. Then, it employs a clustering technique to find groups of biomarkers whose biological processes show a strong interaction. Based on this clustering analysis, this work evaluates how these biological processes influence the overall survival rate in ER+ breast cancer patients, indicating which processes could be predictive of clinical results in response to treatment with TMX. To the best of the authors’ knowledge, this is the first systematic review that presents such a clustering technique for the conjoint evaluation of multiple biomarkers in the survival rate of ER+ breast cancer patients.

## Methodology

### Identifying relevant studies

The manuscript selection was performed according to the PRISMA Extension for Scoping Reviews (PRISMA-ScR) methodology: Checklist and Explanation [23]. The literature review was performed to identify clinical studies assessing the correlation between the expression profile of a molecular biomarker and the long-term outcome of HR+ BC patients in response to TMX treatment. The initial inclusion criteria were original manuscripts, published from 2013/01/01 to 2018/06/30, full texts in any language, obtained from EMBASE or PUBMED databases. For manuscript search, three primary keyword descriptors were applied: “breast cancer”, “hormone therapy” and “resistance”, or their variations listed in Supplementary material 1. Case studies, reviews, and comments were not considered.

### Selection of eligible studies

The manuscripts found in the initial search were analyzed in three stages: first, a title reading was performed, selecting only those presenting all primary keyword descriptors or their variations. Duplicates were removed. The second and third stages consisted of abstract and full-text reading, respectively. These stages ensure that the selected manuscripts respected the inclusion and exclusion criteria, according to Table 1. The essential items of the REMARK protocol [24] were considered in this review. Two independent reviewers performed the manuscript search and selection processes, while a third reviewer solved incongruities. Only manuscripts that assessed molecular biomarkers as a prognostic factor and not as a risk factor were considered. Studies only evaluating patient expression databases, in vivo or in vitro experimentation were also excluded.

**Table 1:** Study inclusion and exclusion criteria

Study characteristics	Inclusion criteria	Exclusion criteria
Design	Cohort Human	Trial Review
Publication	Any language Abstract and full text available	Dissertation Conference proceeding, abstract or poster
Participants	Breast cancer female Hormone therapy	< 35 samples Databases
Intervention	Tamoxifen (TMX)	Hormone therapy non described
Outcome	Correlation with survival or recurrence	Just prognosis with clinical pathological parameters

### Data extraction

After the full-text review stage of the selected manuscripts included in this scoping review, the following information was plotted in Table 2:

- Population: number of participants and follow-up of only those HR+BC patients treated with Tamoxifen.
- Marker profile: the marker symbol used by each selected manuscript; the genetic alteration (deletion, mutation, or polymorphisms) when altered; the type of molecule assessed (DNA, RNA, or protein); the molecular status of the analyzed marker in relation to activity, expression, subcellular localization, or post-translational modification, which could alter its functionality.
- Clinical outcomes and statistical analysis: clinical profile of tested BC patients (survival or recurrence) associated with the alteration of analyzed molecular markers; the statistical analysis of the association between the molecular status of markers and clinical outcome, prioritizing multivariate analysis; the significance level of statistical analysis when available. In order to standardize the collected information, it was plotted molecular profile of markers, which was associated with a poor clinical outcome, except for those presenting polymorphisms or specific subcellular localization.

**Table 2:** Selected publications accessing the influence of molecular markers on clinical outcomes of Tamoxifen-resistant breast cancer patients

Author (Year)	N	Follow-up (years)	Marker symbol	Marker alteration	DNA, RNA or Protein	Marker status	Outcome	Statistical Analysis of Association	*P* value
Browne et al. (2013) [27]	171	12.5	MARCKS	–	Protein	Positive Expression	Poorer OS	Multivariate Analysis	***0.045***
Margolin et al. (2013) [28]	313	13	CYP2D6	–	Protein	Decreased Activity	Poorer RFS	Multivariate Analysis	***0.018***
							Poorer OS	Multivariate Analysis	***0.030***
Zhang et al. (2013) [29]	37	10	NCOR2	BQ323636.1 Splice variant	DNA	Overexpression	Poorer OS	Multivariate Analysis	***0.030***
							Poorer DFS	Multivariate Analysis	***0.038***
Ijichi et al. (2013) [30]	100	16.4	EBAG9	–	Protein	Positive Expression	Poorer DFS	Multivariate Analysis	***0.035***
Piva et al. (2013) [31]	55	8	SOX2	–	Protein	Overexpression	Increased Recurrence	Multivariate Analysis	** < *0.001***
							Poorer DFS	Multivariate Analysis	** < *0.001***
Elzawahry et al. (2013) [32]	70	6	ki-67	–	Protein	Overexpression	Increased recurrence	Multivariate Analysis	***0.007***
Hrstka et al. (2013) [33]	61	10.8	AGR2	–	mRNA	Low Expression	Increased PFS	Univariate Analysis	***0.036***
Chen et al. (2013) [34]	104	10	ERα	Phosphorylation at serine 118	Protein	Phosphorylation at Specific Site	Poorer DFS	Univariate Analysis	***0.022***
							Poorer OS	Univariate Analysis	***0.013***
				Phosphorylation at serine 167	Protein	Phosphorylation at Specific Site	DFS	Univariate Analysis	*0.515*
							OS	Univariate Analysis	*0.300*
Thrane et al. (2014) [35]	244	18–19	AURKA	–	Protein	Overexpression	Poorer DFS	Multivariate Analysis	***0.006***
							Poorer OS	Univariate Analysis	***0.038***
Reijm et al. (2014) [36]	250	3	EZH2	–	Protein	Positive Expression	Poorer PFS	Multivariate Analysis	***0.017***
						% Positive Cells	Poorer PFS	Multivariate Analysis	***0.002***
Huang et al. (2014) [37]	N/A	28	STAT3	–	mRNA	Low Expression	Poorer DMFS	Univariate Analysis	** < *0.001***
					Protein	Low Expression	Poorer DFS	Univariate Analysis	***0.006***
			STAT1	–	Protein	Overexpression	DMFS	Univariate Analysis	*0.067*
							DFS	Univariate Analysis	*0.256*
Wong et al. (2014) [38]	144	26	MAGEA2	–	Protein	Positive Expression	Poorer OS	Univariate Analysis	***0.006***
Putluri et al. (2014) [39]	45	14	RRM2	–	Protein	Overexpression	Poorer RFS	Univariate Analysis	***0.04***
Lehn et al. (2014) [40]	101	16–17	YAP1	–	Protein	Absent Expression	Poorer RFS	Univariate Analysis	** < *0.001***
Wei et al. (2014) [41]	129	6.6	TBK1	–	Protein	Overexpression	Poorer DFS	Univariate Analysis	***0.003***
Winder et al. (2014) [42]	219	10	IGF1R	Polymorphism (rs2016347)	DNA	Presence of Polymorphic Form	Poorer DFS	Multivariate Analysis	***0.024***
							OS	Multivariate Analysis	*0.14*
	*221*	*10*	IGF1	Polymorphism (rs6214)	DNA	Presence of Polymorphic Form	DFS	Multivariate Analysis	*0.95*
							OS	Multivariate Analysis	*0.86*
				Polymorphism (rs7136446)	DNA	Presence of Polymorphic Form	DFS	Multivariate Analysis	*0.74*
							OS	Multivariate Analysis	*0.73*
				Polymorphism (rs2946834)	DNA	Presence of Polymorphic Form	DFS	Multivariate Analysis	*0.58*
							OS	Multivariate Analysis	*0.77*
	*208*	*10*	IGFBP3	Polymorphism (rs2854744)	DNA	Presence of Polymorphic Form	DFS	Multivariate Analysis	*0.58*
							OS	Multivariate Analysis	*0.79*
	*220*	*10*	IRS1	Polymorphism (rs1801123)	DNA	Presence of Polymorphic Form	DFS	Multivariate Analysis	*0.93*
							OS	Multivariate Analysis	*1.0*
Redmond et al. (2014) [43]	N/A	5	HMGB2	–	Protein	Absent Expression	Poorer DFS	Univariate Analysis	***0.006***
Bergamaschi et al. (2014) [44]	501	16	FOXM1	–	Protein	Overexpression	Poorer DFS	Univariate Analysis	***0.003***
Nagelkerke et al. (2014) [45]	304	3	LAMP3	–	mRNA	Overexpression	Poorer PFS	Multivariate Analysis	***0.032***
							Poorer PROS	Univariate Analysis	***0.04***
Karlsson et al. (2015) [46]	73	19	PTPN2	deletion	Protein	Absent Expression	Poorer DRFS	Univariate Analysis	***0.011***
Elias et al. (2015) [47]	76	6–7	FYN	–	Protein	Plasma Membrane-associated Expression	Increased MFS	Univariate Analysis	** < *0.003***
		*12–13*					Increased OS	Univariate Analysis	** < *0.001***
Hato et al. (2015) [48]	335	29	CTSO	Polymorphism (rs10030044)	Protein	Presence of Polymorphic Form	Poorer DFS	Univariate Analysis	***0.005***
							Poorer OS	Multivariate Analysis	** < *0.001***
Honma et al. (2015) [49]	447	20	Bcl-2	–	Protein	Positive Expression	Increased OS	Univariate Analysis	***0.024***
							DFS	Multivariate Analysis	*0.743*
Busch et al. (2015) [50]	87	17	TGFBR2	–	Protein	Low Expression	Poorer RFS	Univariate Analysis	***0.008***
	*99*	17	SMAD2	Phosphorylation at serine 465/467	Protein	Phosphorylation at specific site	RFS	Univariate Analysis	*0.878*
Larsen et al. (2015) [51]	259	19	SRC	–	Protein	Plasma Membrane-associated Expression	Poorer DFS	Multivariate Analysis	***0.004***
							Poorer OS	Multivariate Analysis	***0.026***
Argalácsová et al. (2015) [52]	71	16–17	CYP2D6	Several polymorphisms	Protein	Enzymatic Activity	DFS and TTP	Univariate Analysis	*N/A*
			ABCB1	Polymorphism (rs2032582)	DNA	Presence of Polymorphic Form	DFS	Univariate Analysis	*N/A*
				Polymorphism (rs1045642)	DNA	Presence of Polymorphic Form	Increased DFS	Univariate Analysis	***0.012***
Bentin et al. (2015) [53]	94	36	AKAP13	–	mRNA	Overexpression	Poorer PFS	Multivariate Analysis	***0.044***
Zhong et al. (2016) [54]	88	12	FRS2	–	Protein	Overexpression	Poorer DFS	Multivariate Analysis	***0.03***
			miR-4653-3p	–	miRNA	Low Expression	Poorer DFS	Multivariate Analysis	***0.004***
Ahern et al. (2016) [55]	911	–	Pak1	–	Protein	Cytoplasmic Expression Level	Recurrence	Univariate Analysis	*N/A*
	962					Nuclear Expression	Recurrence	Univariate Analysis	*N/A*
Babyshkina et al. (2016) [56]	97	8–9	EGFR	–	Protein	Positive Expression	Poorer PFS	Univariate Analysis	***0.006***
Liu et al. (2016) [57]	148	2.9	GATA3	Mutation	Protein	Presence of Mutated Form	PFS	Univariate Analysis	*0.800*
				–	mRNA	Low Expression	ORR	Univariate Analysis	*0.31*
							Poorer PFS	Multivariate Analysis	***0.008***
De Marchi et al. (2016) [58]	317	3	ANXA1	–	Protein	Overexpression	Shorter TTP	Multivariate Analysis	***0.016***
	259	3	CALD1	–	Protein	Overexpression	Shorter TTP	Multivariate Analysis	***0.001***
De Marchi et al. (2016) [59]	294	3	PDCD4	–	Protein	Overexpression	Longer TTP	Multivariate Analysis	***0.009***
			OCIAD1	–	Protein	Expression	TTP	Univariate Analysis	*N/A*
			CGN	–	Protein	Expression	TTP	Univariate Analysis	*N/A*
			G3BP2	–	Protein	Expression	TTP	Univariate Analysis	*N/A*
Sensorn et al. (2016) [60]	73	14.3	ABCC2	Polymorphism (rs717620)	DNA	Presence of Polymorphic Form	Increased DFS	Multivariate Analysis	***0.040***
Bekele et al. (2016) [71]	94	8	Nrf2	–	Protein	Overexpression	Poorer OS	Univariate Analysis	***0.002***
			ABCC1	–	Protein	Overexpression	Poorer OS	Univariate Analysis	***0.04***
			ABCC3	–	Protein	Overexpression	Poorer OS	Univariate Analysis	***0.01***
			NQO1	–	Protein	Expression	OS	Univariate Analysis	*0.12*
Van der Willik et al. (2016) [61]	245	3	SIAH2	–	Protein	Overexpression	Poorer PFS	Multivariate Analysis	***0.015***
Thistle et al. (2017) [62]	1082	10	14–3-3ζ	–	Protein	Nuclear and Cytoplasmic Overexpression	Poorer RFS	Univariate Analysis	***N/A***
Gwak et al. (2017) [63]	129	10.5	Oct4	–	Protein	Overexpression	Poorer DFS	Multivariate Analysis	** < *0.001***
Abudureyimu et al. (2017) [64]	76	13–14	AURKB	–	Protein	Positive Expression	Poorer OS	Univariate Analysis	***0.001***
Snell et al. (2017) [65]	63	10	PR	–	Protein	Absent Expression	Poorer RFS	Multivariate Analysis	***0.005***
Baldacchino et al. (2017) [66]	187	16–20	CIP2A	–	Protein	Plasma Membrane-associated Expression	DFS	Multivariate Analysis	***0.002***
Han et al. (2017) [67]	95	12	miR-222	–	miRNA	Expression	DFS	Univariate Analysis	*0.286*
Zhang et al. (2018) [68]	291	3	PAK2	–	Protein	Overexpression	Poorer PFS	Multivariate Analysis	***0.008***
	101			–	mRNA	Overexpression	Poorer PFS	Univariate Analysis	***0.033***
Moon et al. (2018) [69]	99	12.9	CD24	–	Protein	Positive Expression	Poorer PFS	Multivariate Analysis	***0.006***
			CD44	–	Protein	Expression	PFS	Univariate Analysis	*1.0*
			ALDH1	–	Protein	Expression	PFS	Univariate Analysis	*0.090*
Choi et al. (2018) [70]	85	12.5	RBP2	–	Protein	Overexpression	Poorer DFS	Univariate Analysis	***0.04***

The results in bold italic refers to a significant p value (p < 0.05). Meanwhile, the italic font illustrates all results without a significant p value, i.e., they do not obey the p < 0.05 criteria

OS: Overall survival; DFS: disease-free survival; RFS: relapse-free survival; PFS: progression-free survival; DMFS: distant metastasis free survival; PROS: post-relapse free survival; DRFS: distant recurrence free survival; MFS: metastasis free survival; TTP: time to progression; ORR: overall response rate

### In silico analysis

#### Design of networks

After a full-length analysis of manuscripts and data extraction, the official symbol of all selected molecular markers was confirmed in the HUGO Gene Nomenclature Committee (HGNC) website for in silico analysis (Supplementary Table 1). Those presenting statically significant associations (p < 0.05) with some clinical outcomes in HR+ BC patients were assessed together using the ©STRING CONSORTIUM 2019 (version 11.0) web tool. The minimum interaction score adopted in this analysis is 0.700 (high confidence) for each connection (edges), including all active interaction sources used by the tool, excluding text mining. The resulted network was presented with an interaction score of each connection between two markers for further modular analysis of networks. Posteriorly, the unconnected markers were separately analyzed. Only those markers whose molecular profile of expression/activity direct influence the clinical outcomes of HR+ BC patients were considered, excluding micro RNAs. For polymorphisms, only the affected biomarker was included for network design, not considering the polymorphic variant itself.

#### Modular analysis of network

From the STRING resulting network, it was used ClusterONE plugin [25] of Cytoscape software. The cluster establishment was assessed considering the following criteria: at least three proteins compounding a cluster, density and interaction quality values above 0.5, and significant p-value (p < 0.05). Molecular markers that were not included in any cluster were called unclustered markers.

#### Gene ontology analysis

Once the clusters within a molecular marker network were identified, it was performed the gene ontology (GO) analysis using the Biological Network Gene Ontology (BiNGO) plugin (version 3.0.3) in Cytoscape software to assess the possible biological processes that could be involved with the specific set of markers grouped in each cluster. Statistically significant clusters were assessed, considering only their members or added to their direct neighbor markers (networked markers directly linked with clustered markers). Statistical significance was measured using a hypergeometric test with the multiple testing correction of Benjamini & Hochberg False Discovery Rate (FDR). Only biological processes with a p-value < 0.05 were considered significant. Based on biological knowledge about BC resistance, the five biological processes often involved with this event were selected to be illustrated in the results and further discussed.

#### The predictive power of different sets of TMX-resistance markers 

In order to assess the predictive power of each set of markers among those significantly associated with any clinical outcome in TMX-treated HR+ BC patients, the *Kaplan Meier-plotter web software* [26] was used. It was assessed a general analysis for all selected markers, those connected and unconnected markers from STRING analysis, and those clusters obtained from ClusterOne plugin (with or without their direct neighbor markers). The data extracted from the original papers showing high expression/activity of molecular markers associated with poor outcome were directly related in the KM-plotter analysis settings, inverting those presenting low or absence expression/activity associated with poor prognosis status (Supplementary Table 1). Equal weights were attributed to all tested markers. The Kaplan–Meier graphs represent overall survival (OS) with a follow-up threshold of 240 months, splitting patients by median, using all probe sets per gene, without any restriction of breast cancer subtypes or selected cohorts studies as dataset source.

## Results

**Fig. 1:**
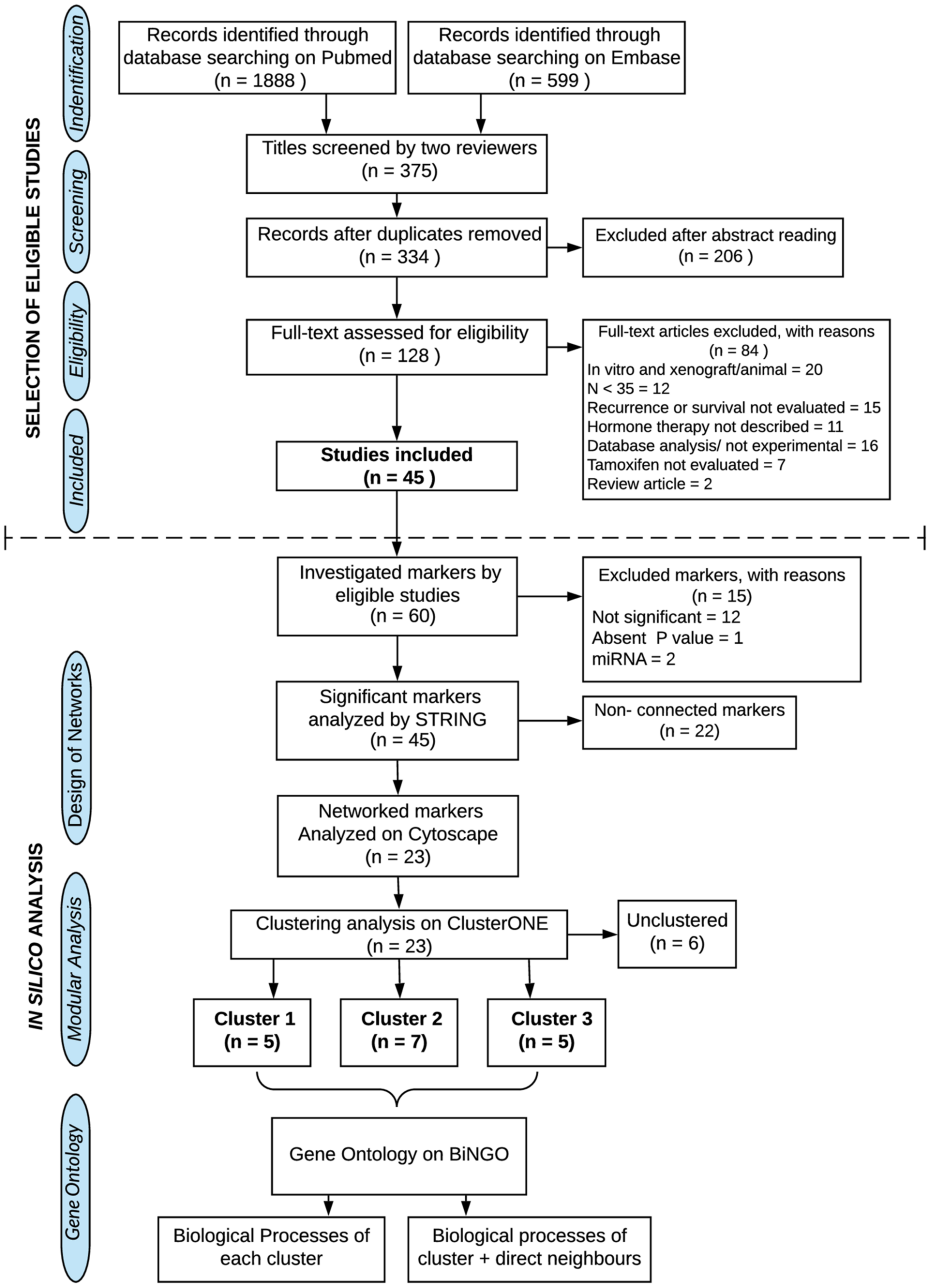
PRISMA diagram of selection of studies and in silico analysis. The upper section corresponds to the step-by-step manuscript selection process according to the established inclusion and exclusion criteria. The Lower section includes the sequence of in silico analysis performed with all selected biomarkers to search a better and integrated understanding of the influence of each protein in TMX resistance

It was identified 2.487 articles in EMBASE and PUBMED databases from the keyword descriptors and their variations. The flow diagram of literature analysis, manuscript selection, and in silico analysis is illustrated in Fig. 1.

The application of the eligibility criteria resulted in the exclusion of 2.443 papers that were not adequate to one or more selection factors or did not provide the needed information. The main excluding factors were: (1) in vitro experimentation; (2) in vivo assays; (3) clinical studies assessing less than 35 patients (4) the absence of prognostic factor assessing in clinical studies; (5) the absence of hormone therapy with TMX; (6) manuscripts using of transcription or genomic sequencing data deposited in public databases; (7) works testing other types of hormone therapy, differently of TMX; (8) and review manuscripts. Thereby, 45 manuscripts were selected from the eligibility analysis, analyzing 60 different molecular targets, as shown in Table 2.

The total number of analyzed patients in each selected study has varied between 37 [29] and 1082 [62]. The follow-up of most studies was 5 years or more, and 6 other studies presented a follow-up of 3 years [36, 45, 58, 59, 61, 68] or less [57]. The main reported alterations in assessed molecular targets were changes in their expression level or activity. However, some structural modifications at the DNA, RNA, or protein level were also found, such as polymorphisms, post-translational modifications (phosphorylation), and mutations (deletion or splicing variant).

**Fig. 2:**
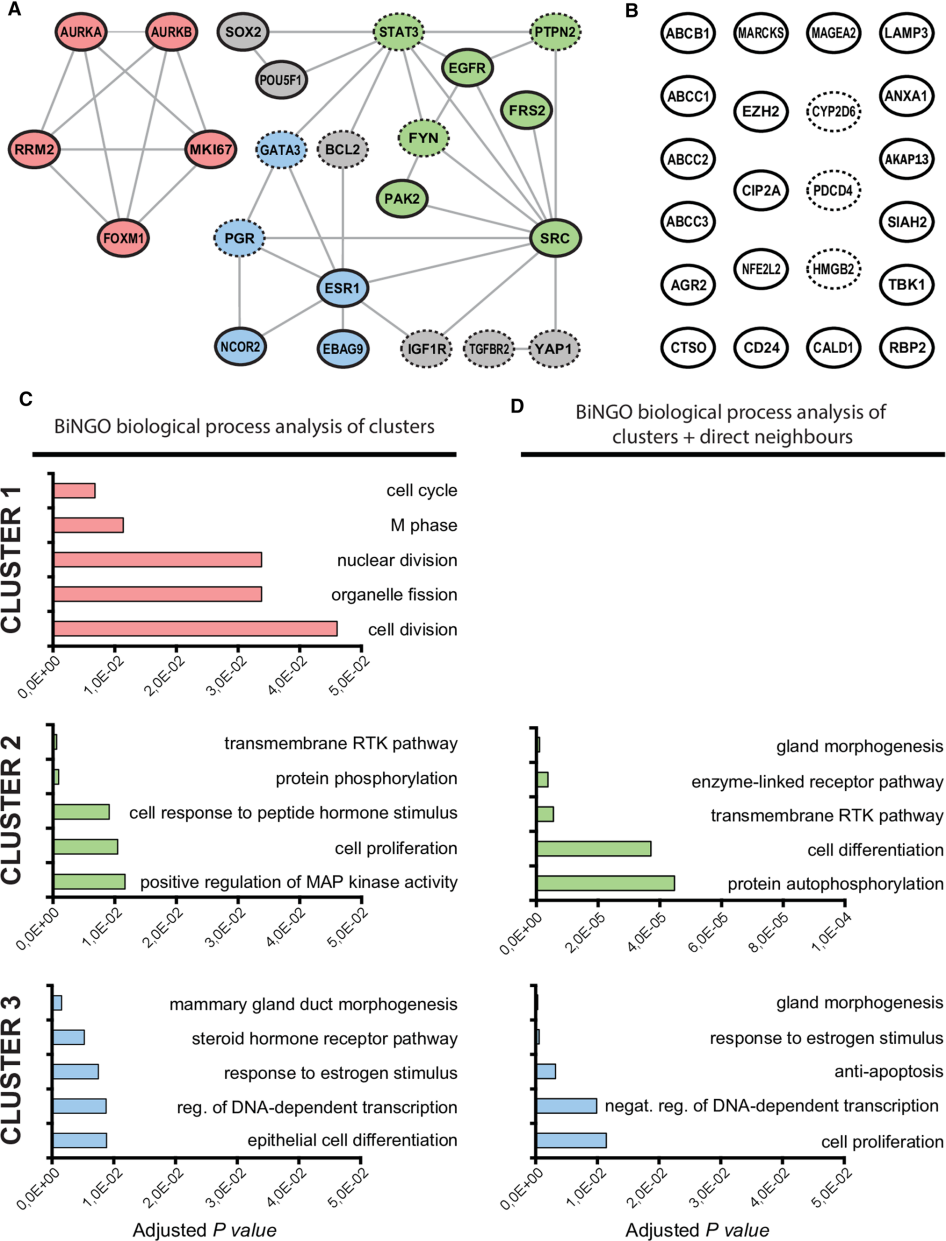
In silico analysis. **A** 3 clusters were formed with strongly linked molecular markers, cluster 1(red), cluster 2 (green), and cluster 3 (blue). The markers in gray were not included in the clusters, remaining just interconnected proteins among clusters inside the network. **B** unconnected markers. A and B still reveal the functional status of each molecular marker. 12 markers (dashed borderline in the molecular markers) decrease in functional activity associated to poor outcomes in BC patients, the other 33 markers presented an elevated functional activity associated to worst outcomes (full borderline in the molecular markers), accordingly information of their original manuscripts. **C** Gene ontology analysis for biological processes: CLUSTER 1 presented significant involvement with molecular events related to cell proliferation, CLUSTER 2 with the modulation of proliferative mechanisms, and CLUSTER 3 with the mammary gland development and its hormone stimulation. **D** Genetic ontology analysis of clusters 2 and 3, taking into account not only its constituent markers but with its direct neighbors

Fifteen of the 60 selected markers were excluded from the in silico analysis (Fig. 1). Thereby, only 45 of 60 selected markers were eligible for in silico analysis. STRING analysis resulted in a functional association among 23 of 45 tested markers, forming two networks with 5 and 18 nodes, respectively (Fig. 2a).

Furthermore, to find densely associated subgroups of molecular markers inside the obtained networks, ClusterONE analysis revealed the presence of 3 clusters with strongly linked molecular markers (red, green, and blue markers). Five proteins (AURKA, MK167, AURKB, FOXM1, RRM2) constituted the CLUSTER 1 (red markers) that presented a density value = 1,000 and interaction quality = 1000, displaying a high interactive power among these markers, represented by a *p-value* = 0.002. The CLUSTER 2 (green markers) was configured with 7 proteins (STAT3, PTPN2, FYN, SRC, PAK2, FRS2, EGFR), presenting a density value = 0.571 and quality = 0.600 and a *p-value* = 0.022. The CLUSTER 3 (blue markers) was established with 5 proteins (ESR1, GATA3, PGR, NCOR2, EBAG9), presenting density = 0.600, interaction quality = 0.545 and *p-value* = 0.055, considered a borderline, accordingly the established criteria.

Not all networked molecular markers were included in the clusters, remaining just interconnected proteins among clusters inside the network (gray markers). This subgroup was called unclustered markers and was constituted by six proteins (SOX2, POU5F1, BCL2, IGF1R, TGFBR2, YAP1). Among these, SOX2 and POU5F1 were directly linked only with CLUSTER 2, and TGFBR2 and YAP1 only with CLUSTER 3. On the other hand, BCL2 and IGF1R seem to establish a link between CLUSTER 2 and 3, acting as an intermediate node between them. The other remaining 22 markers presented no interaction among them (Fig. 2b).

Figure 2a and 2b reveal the functional status of each molecular marker that is associated with a worsening outcome in patients, according to the information of their original manuscripts. From 45 tested markers, 12 of them presented a decrease in functional activity associated with poor outcomes in BC patients (dashed borderline in the molecular markers), which may be due to an inactivating polymorphism, a gene deletion, a gene/protein expression decrease, or still an activity reduction. Further, the other 33 markers presented an elevated functional activity associated with the worst outcomes due to an activating polymorphism, elevated gene/protein expression, or increasing functional activity.

In the gene ontology analysis (Fig. 2c), CLUSTER 1 presented significant involvement with molecular events related to cell proliferation, strongly suggesting that this subset of molecular markers could directly influence the mitotic mechanism regulation. The analysis of CLUSTER 2 presented significant implications in biological processes directly connected with signaling pathways involved with the modulation of proliferative mechanisms. Lastly, CLUSTER 3 showed a significant relationship with processes related to the development of the mammary gland and its hormone stimulation, suggesting a possible involvement with mechanisms of differentiation and signaling pathways of sexual hormone response in the breast tissue.

CLUSTER 2, together with its direct neighbors (BCL2, SOX, POU5F1, GATA3, YAP1. ESR1, PGR, IGF1R) (Fig. 2d), has shown significant implication with biological processes as “gland morphogenesis,” “enzyme-linked receptor pathway,” “transmembrane RTK pathway,” “cell differentiation” and “protein autophosphorylation.” The same approach was followed for CLUSTER 3 and its direct neighbors (BCL2, STAT3, SRC, IGF1R), resulting in significant involvement with cellular mechanisms such as “gland morphogenesis,” “response to estrogen stimulus,” “anti-apoptosis,” “negative regulation of DNA-dependent transcription” and “cell proliferation.” It was found that some cell processes had changed between CLUSTER 2 and 3 when direct neighbors were added in analysis, highlighting their proximity inside the network and the possible involvement of both clusters in similar or the same cellular processes.

**Fig. 3:**
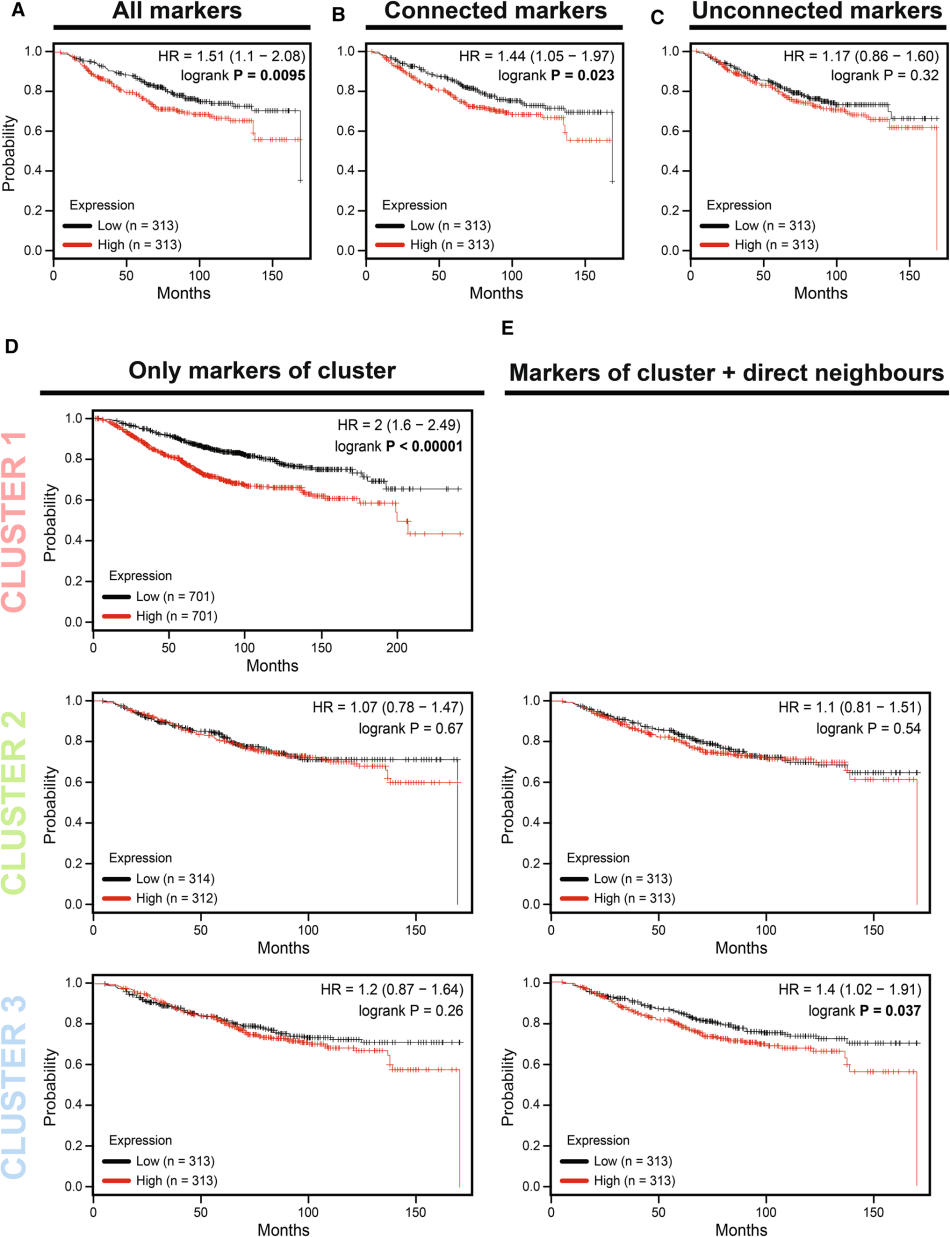
Predictive analysis of overall survival for each set of markers in BC patients using the Kaplan–Meier plotter tool. **A** Analysis of all 45 selected markers. **B** Only the networked markers (23 markers). **C** Unconnected markers (22 markers). **D** Analysis of clusters 1, 2, and 3. **E** Cluster 2 and 3 with their direct neighbors. Significant p-values (< 0.05) are highlighted in bold

In order to complement our data, a more in-depth analysis was carried out to test if these clusters would have a predictive power of overall survival in BC patients when treated with TMX (Fig. 3).

For this analysis, *Kaplan Meier-plotter web software* [26] was used, assessing the overall survival (OS) with a follow-up of 240 months. A first analysis of all 45 selected markers shown hazard ratio (HR) 1.51 (95% CI 1.1–2.08) and a p-value = 0.0095 (Fig. 3a). A significant predictive power was found when only those networked markers were analyzed, with an HR 1.44 (95% CI 1.05–1.97), p-value = 0.023 (Fig. 3b). As expected, the joint analysis of non-connected markers was not significant in predicting overall survival (Fig. 3c).

Furthermore, it was assessed if the specific molecular clusters would present predictive power for overall survival. Figure 3d shows Kaplan–Meier for each analyzed cluster, and, among them, only CLUSTER 1 has presented significant predictive capability, displaying HR = 2.0 (95% CI 1.6–2.49), p-value < 0.00001. Conversely, knowing the proximity and possible functional overlap between CLUSTER 2 and 3, it was assessed the predictive power of specific clusters together with their direct neighbors (Fig. 3e). Only CLUSTER 3 with its direct neighbors displayed significant results, with HR 1.4 (95% CI 1.02–1.91), p-value = 0.037.

## Discussion

This review found evidence in the literature about possible molecular targets capable of individually predicting TMX resistance in patients with BC, demonstrating a strong association between their molecular status and specific clinical outcome, such as tumor survival and/or recurrence. Therefore, an in silico analysis was performed to aid in data analysis. An interrelation between some molecular targets was found, forming three distinct clusters, showing significant involvement in biological processes. Our method, based on a dense analysis from literature datasets, showed that there might be groups of cellular processes that could be more involved with resistance to TMX in HR+BC patients. It is worth highlighting that the same weight was assigned for all analyzed markers in the KM-plot analysis since this information is not commonly evaluated in the study models of the original data sources. Therefore, it might represent a limitation in the present analysis since each marker can have different influence degrees within a signaling pathway to control a specific biological process.

The markers MKI67, AURKA, AURKB, FOXM1 in CLUSTER 1 presented significant involvement with molecular events related to cell proliferation (Fig. 2c), as observed by their original manuscripts [32, 35, 39, 44, 64]. However, this cellular process is highly complex, involving several components that could regulate it positively or negatively, and it has been widely studied as it is crucial for resistant subpopulation rising, not only to TMX but also to other drugs [72–75].

Analysis of overall survival of CLUSTER 1 showed a significant association with a worse outcome of the TMX-treated BC patients (Fig. 3d) when strongly expressed or activated. Bergamaschi et al*.* showed that the analysis of a gene signature related to mitosis control, orchestrated by marker 14-3-3ζ, was able to control the resistance profile to endocrine therapy in BC, influencing the expression of markers involved with CLUSTER 1, such as FOXM1 and AURKB [76]. Perhaps, if the *Thistle JE. *et al*.*, who analyzed the involvement of 14-3-3ζ with a worse outcome in TMX+ treated ER+ patients, had presented statistical correlation values, this marker would be highly related to CLUSTER1 and could result in an increase of predictive power for this set of genes for TMX therapy response [62].

The uncontrolled cell cycle is considered a hallmark of cancer [77], as shown in the meta-analysis that evaluated three key biological processes for the development of breast cancer (cell proliferation, ER, and HER2 signaling). This analysis highlighted cell proliferation as an important prognostic role. Further, the results showed that the subtypes ER+/HER2- with low proliferation showed a higher DRFS, whereas the ER−/HER2− and HER2+ showed a higher level of cell proliferation and worse outcomes. However, there was no analysis of the markers with regard to the different hormonal treatments [21].

Besides, there are studies providing information about possible combined effects among these markers associated with a worse prognosis in patients with other BC subtypes [78, 79]. However, no studies simultaneously evaluate the expression profile of five markers composing CLUSTER 1 in tumor cells, which is a promising molecular target to be better explored in future research about this predictive power in response to TMX treatment.

No significant correlation was found with the clinical outcomes for CLUSTERS 2 and 3 (considering only the constituent markers) (Fig. 3d). However, individually, each molecular marker has presented an association with worse outcomes in their original studies. This controversial effect of joint analysis demonstrates the importance of simultaneously analyzing as many markers as possible and jointly correlating with clinical outcomes. The more extensive and more complete the molecular profile of tumor cells is, the closer to reality this analysis will be, increasing the representativeness of the experimental data.

Reinforcing this concept, when only constituting markers of CLUSTER 3 were analyzed, no association was found with some clinical outcome. However, when other close markers were included in this analysis, a significant association with overall survival was observed (Fig. 3e). The combination of CLUSTER 3 and its direct neighbors have shown involvement with cellular processes linked to mammary gland development and differentiation, in addition to sexual hormone response and gene transcription regulation (Fig. 2d). The latter has been associated with Decitabine (DAC) chemoresistance, also in BC [80]. However, some of these cellular processes are quite generic and could be related to several molecular markers. Therefore, more evidence about this subset of markers is required to increase the number of molecular components integrating this cluster, restrict the involvement with generic cellular processes, and improve its predictive power concerning clinical outcomes.

The investigation of gene signatures relating a specific cell profile with specific clinical outcomes has gained traction, not only for BC but also for other tumor types. Toshimitsu et al. revealed a specific molecular profile of cisplatin resistance in esophageal cancer based on in vitro studies using drug-resistant cell lineage [81]. Other works have discovered gene signatures related to BC resistance for the most varied treatments, such as neoadjuvant therapy [82], inhibitory molecules [83], and hormone therapy [84], among others. These studies make use of multiple strategies, such as transcriptome sequencing and microarray, to simultaneously investigate a range of markers in the same tumor sample and compare it with the resistant condition.

The study used a gene expression assay to quantify the likelihood of recurrence in patients with positive breast cancer, negative nodule, and ER+ treated with TMX. The study managed to identify 16 genes that were related to cancer recurrence that belonged to either of the following biological processes: proliferation (such as Ki-67 and cyclin B1), estrogen receptor (including genes such as ER and PR), HER2 (including HER2 and GRB7), and cell invasion (including genes like Stromelysin 3 and Cathepsin L2) [85]. A follow-up study was developed to investigate the ESR1 marker, which has been reported as a strong predictor of resistance to TMX in ER-positive patients with low levels of ESR1 [86].

Mihály et al. performed a meta-analysis looking for studies that evaluated expression-based markers to provide independent information regarding TMX treatment. Out of 68 biomarkers with probable connection to the TMX resistance, three (PGR, MAPT, and SLC7A5) were the most promising observed in patients treated with TMX. However, only independent markers were evaluated without making a joint analysis to investigate their main biological processes. According to our review, we can also observe that many markers remain under study [22].

In conclusion, this systematic review is the first of its kind to adopt a clustering technique to evaluate the independent data on the literature regarding TMX resistance in ER+ breast cancer patients. This analysis revealed three networks of biomarkers involved in biological processes: cell cycle, signal transduction of proliferative stimuli, and hormone response involved in morphogenesis and differentiation of mammary gland. As expected, CLUSTER 1 corroborated that the cell proliferation pathway is strongly linked to the prediction of TMX response. Further, CLUSTER 2 and 3 indicate that there are other resistance pathways that must be thoroughly investigated as currently available data are not sufficient to reach a statistically significant conclusion. When the direct cluster neighbors are considered, CLUSTER 3 showed a strong link between TMX resistance and the development of the mammary gland and its hormone stimulation. Thus, our data found are hypothesis generators, which suggest promising mechanisms and biomarkers for predicting resistance to TMX and contributing to future studies and personalized medicine.

## Supplementary Information

Additional file1 (DOCX 13 KB)

Additional file2 (DOCX 26 KB)

## Data Availability

The datasets used and/or analysed during the current study are available from the corresponding author on reasonable request.

## Abbreviations

TMX: Tamoxifen
ER+: Estrogen of receptor-positive
BC: Breast cancer
HR+: Hormone receptor expression
PR+: Progesterone receptor
SERM: Selective estrogen receptor modulator
REMARK: Reporting recommendations for tumor marker prognostic
GO: Gene ontology
BiNGO: Biological Network Gene Ontology
FDR: False discovery rate
OS: Overall survival
AURKA: Aurora kinase A
MK167: Marker of proliferation Ki-67
AURKB: Aurora kinase B
FOXM1: Forkhead box M1
RRM2: Ribonucleotide reductase regulatory subunit M2
STAT3: Signal transducer and activator of transcription 3
PTPN2: Protein tyrosine phosphatase non-receptor type 2
FYN: Protein-tyrosine kinase oncogene family
SRC: Proto-oncogene, non-receptor tyrosine kinase
PAK2: Serine/threonine protein kinase
FRS2: Fibroblast growth factor receptor substrate 2
EGFR: Epidermal growth factor receptor
ESR 1: Estrogen receptor 1
GATA3: Transcription factor
PGR: Progesterone receptor
NCOR2: Nuclear receptor corepressor 2
EBAG9: Estrogen receptor binding site associated antigen 9
SOX2: SRY-box transcription factor 2
POU5F1: POU class 5 homeobox 1
BCL2: B-cell lymphoma 2
IGF1R: Insulin like growth factor 1 receptor
TGFBR2: Transforming growth factor beta receptor 2
YAP1: Yes-associated protein 1
