# Supplementary material for: Molecular markers associated with the outcome of tamoxifen treatment in estrogen receptor-positive breast cancer patients: scoping review and in silico analysis
Source: Discov Oncol. 2021 Oct 1;12:37. doi: 10.1007/s12672-021-00432-7 (PMC8777552; doi:10.1007/s12672-021-00432-7)
Supplement: Supplementary file 1 — Additional file1 (DOCX 13 KB) [file 12672_2021_432_MOESM1_ESM.docx]

Synonym search descriptors:

((“Breast Cancer” or “Breast Neoplasm” or "Neoplasm, Breast" or "Breast Tumors" or "Breast Tumor" or "Tumor, Breast" or "Tumors, Breast" or "Neoplasms, Breast" or "Breast Carcinoma" or "Breast Carcinomas" or "Luminal Carcinoma Breast" or "Carcinoma, Breast" or "Carcinomas, Breast" or "Mammary Neoplasms, Human" or "Human Mammary Neoplasm" or "Human Mammary Neoplasms" or "Neoplasm, Human Mammary" or "Neoplasms, Human Mammary" or "Mammary Neoplasm, Human" or "Cancer, Breast Mammary Cancer" or "Cancer, Mammary" or "Cancers, Mammary" or "Mammary Cancers" or "Malignant Neoplasm of Breast" or "Breast Malignant Neoplasm" or "Breast Malignant Neoplasms" or "Malignant Tumor of Breast" or "Breast Malignant Tumor" or "Breast Malignant Tumors" or "Cancer of Breast" or "Cancer of the Breast") and (“hormone therapy” or “estrogen receptor” or “estrogen receptor positive” or “hormonal therapy” or “luminal” or “ER+” or “ER positive” or “tamoxifen” or “Nolvadex” or “Novaldex” or “Tamoxifen Citrate” or “Citrate, Tamoxifen” or “Tomaxithen” or “Zitazonium” or “Soltamox”) and ("Resistance” or “Locoregional Neoplasm Recurrence” or “Local Neoplasm” or “Neoplasm Recurrences, Local” or “Recurrence, Local Neoplasm” or “Recurrence, Locoregional Neoplasm” or “Local Neoplasm Recurrence” or “Neoplasm Recurrence, Locoregional” or “Locoregional Neoplasm Recurrences” or “Neoplasm Recurrences, Locoregional” or “Recurrences, Locoregional Neoplasm” or “Resistance, drug” or “Multiple Drug Resistance” or “Resistance, Multiple Drug” or “Multidrug Resistance” or “Multi-Drug Resistance” or “Local Neoplasm Recurrences” not "review"))
