# Supplementary material for: Molecular markers associated with the outcome of tamoxifen treatment in estrogen receptor-positive breast cancer patients: scoping review and in silico analysis
Source: Discov Oncol. 2021 Oct 1;12:37. doi: 10.1007/s12672-021-00432-7 (PMC8777552; doi:10.1007/s12672-021-00432-7)
Supplement: Supplementary file 2 — Additional file2 (DOCX 26 KB) [file 12672_2021_432_MOESM2_ESM.docx]

| **Supplementary Table 1.** Analyzed markers in the selected manuscripts and its stratification according to the inclusion/ exclusion criteria for further analysis | | | | | | | | | | |  |  |
| --- | --- | --- | --- | --- | --- | --- | --- | --- | --- | --- | --- | --- |
| **Inclusion/exclusion**  **Criteria of Markers** | | **Marker Names** | | | **HGNC ID** | | | **Official Marker Name** | **Relation with**  **poor outcome for**  **KM-plot Analysis** | |  |  |
| **Included markers** | |  | | |  | | |  |  | |  |  |
| **Significant** correlation with outcome in TMX-treated RE+ breast cancer patients | | ABCB1 | | | 40 | | | ABCB1 | Direct | |  |  |
|  |  | ABCC1 | | | 51 | | | ABCC1 | Direct | |  |  |
|  |  | ABCC2 | | | 53 | | | ABCC2 | Direct | |  |  |
|  |  | ABCC3 | | | 54 | | | ABCC3 | Direct | |  |  |
|  |  | AGR2 | | | 328 | | | AGR2 | Direct | |  |  |
|  |  | AKAP13 | | | 371 | | | AKAP13 | Direct | |  |  |
|  |  | ANXA1 | | | 533 | | | ANXA1 | Direct | |  |  |
|  |  | AURKA | | | 11393 | | | AURKA | Direct | |  |  |
|  |  | AURKB | | | 11390 | | | AURKB | Direct | |  |  |
|  | | BCL-2 | | | 990 | | | BCL2 | Inverse | |  |  |
|  | | CALD1 | | | 1441 | | | CALD1 | Direct | |  |  |
|  | | CD24 | | | 1645 | | | CD24 | Direct | |  |  |
|  | | CIP2A | | | 29302 | | | CIP2A | Direct | |  |  |
|  | | CTSO | | | 2542 | | | CTSO | Direct | |  |  |
|  | | CYP2D6^a^ | | | 2625 | | | CYP2D6 | Inverse | |  |  |
|  | | EBAG9 | | | 3123 | | | EBAG9 | Direct | |  |  |
|  | | EGFR | | | 3236 | | | EGFR | Direct | |  |  |
|  | | ERα | | | 3467 | | | ESR1 | Direct | |  |  |
|  | | EZH2 | | | 3527 | | | EZH2 | Direct | |  |  |
|  | | FOXM1 | | | 3818 | | | FOXM1 | Direct | |  |  |
|  | | FRS2 | | | 16971 | | | FRS2 | Direct | |  |  |
|  | | FYN | | | 4037 | | | FYN | Inverse | |  |  |
|  | | GATA3 | | | 4172 | | | GATA3 | Inverse | |  |  |
|  | | HMGB2 | | | 5000 | | | HMGB2 | Inverse | |  |  |
|  | | IGF1R | | | 5465 | | | IGF1R | Inverse | |  |  |
|  | | Ki-67 | | | 7107 | | | MKI67 | Direct | |  |  |
|  | | LAMP3 | | | 14582 | | | LAMP3 | Direct | |  |  |
|  | | MAGEA2 | | | 6800 | | | MAGEA2 | Direct | |  |  |
|  | | MARCKS | | | 6759 | | | MARCKS | Direct | |  |  |
|  | | NCOR2 | | | 7673 | | | NCOR2 | Direct | |  |  |
|  | | Nrf2 | | | 7782 | | | NFE2L2 | Direct | |  |  |
|  | | Oct4 | | | 9221 | | | POU5F1 | Direct | |  |  |
|  | | PAK2 | | | 8591 | | | PAK2 | Direct | |  |  |
|  | | PDCD4 | | | 8763 | | | PDCD4 | Inverse | |  |  |
|  | | PR | | | 8910 | | | PGR | Inverse | |  |  |
|  | | PTPN2 | | | 9650 | | | PTPN2 | Inverse | |  |  |
|  | | RBP2 | | | 9920 | | | RBP2 | Direct | |  |  |
|  | | RRM2 | | | 10452 | | | RRM2 | Direct | |  |  |
|  | | SIAH2 | | | 10858 | | | SIAH2 | Direct | |  |  |
|  | | SOX2 | | | 11195 | | | SOX2 | Direct | |  |  |
|  | | SRC | | | 11283 | | | SRC | Direct | |  |  |
|  | | STAT3 | | | 11364 | | | STAT3 | Inverse | |  |  |
|  | | TBK1 | | | 11584 | | | TBK1 | Direct | |  |  |
|  | | TGFBR2 | | | 11773 | | | TGFBR2 | Inverse | |  |  |
|  | | YAP1 | | | 16262 | | | YAP1 | Inverse | |  |  |
| **Excluded markers** |  | | |  |  |  |  |  |  |  |  |  |
| **No significant** correlation with outcome in TMX-treated RE+ breast cancer | | ALDH1 | | | | 402 | | ALDH1A1 |  | | |  |
|  |  | CD44 | | | | 1681 | | CD44 |  | | |  |
|  |  | CGN | | | | 17429 | | CGN |  | | |  |
|  | | CYP2D6^a^ | | | | 2625 | | CYP2D6 |  | | |  |
|  | | G3BP2 | | | | 30291 | | G3BP2 |  | | |  |
|  | | IGF1 | | | | 5464 | | IGF1 |  | | |  |
|  | | IGFBP3 | | | | 5472 | | IGFBP3 |  | | |  |
|  | | IRS1 | | | | 6125 | | IRS1 |  | | |  |
|  | | NQO1 | | | | 2874 | | NQO1 |  | | |  |
|  | | OCIAD1 | | | | 16074 | | OCIAD1 |  | | |  |
|  | | PAK1 | | | | 8590 | | PAK1 |  | | |  |
|  | | SMAD2 | | | | 6768 | | SMAD2 |  | | |  |
|  | | STAT1 | | | | 11362 | | STAT1 |  | | |  |
|  | |  | | | | |  |  |  |  |  |  |
| Marker without *p value* | | 14-3-3ζ^b^ | | | | 12855 | | YWHAZ |  | | |  |
| Micro RNAs | | miR-222 | | | | 31602 | | hsa-miR-222-3p^c^ |  | | |  |
|  | | | miR-4653-3p | | 41562 | | | hsa-miR-4653-3p^d^ | |  | | |

^a^CYP2D6 was analyzed in two different manuscripts presenting both significant and no significant correlation with outcome in RE+ breast cancer patients after TMX treatment.

^b^14-3-3ζ was shown a supposed correlation with outcome in RE+ breast cancer patients treated with TMX, but the original manuscript did not show the *p value* to confirm statistical significance.

^c^MIMAT0000279 is the accession code in miRBase (www.miRBase.org; accessed in may 25, 2019)

^d^MIMAT0019719 is the accession code in miRBase (www.miRBase.org; accessed in may 25, 2019)
